# Freezing African Elephant Semen as a New Population Management Tool

**DOI:** 10.1371/journal.pone.0057616

**Published:** 2013-03-06

**Authors:** Robert Hermes, Joseph Saragusty, Frank Göritz, Paul Bartels, Romain Potier, Barbara Baker, W. Jürgen Streich, Thomas B. Hildebrandt

**Affiliations:** 1 Department of Reproduction Management, Leibniz Institute for Zoo and Wildlife Research, Berlin, Germany; 2 NZG Biobank, National Zoological Gardens of South Africa, National Research Foundation, Pretoria, Republic of South Africa; 3 ZooParc de Beauval, Saint Aignan sur Cher, France; 4 Pittsburgh Zoo and PPG Aquarium, Pittsburgh, Pennsylvania, United States of America; Federal University of Parana (UFPR) ) – Campus Palotina, Brazil

## Abstract

**Background:**

The captive elephant population is not self-sustaining and with a limited number of breeding bulls, its genetic diversity is in decline. One way to overcome this is to import young and healthy animals from the wild. We introduce here a more sustainable alternative method - importation of semen from wild bulls without removing them from their natural habitat. Due to the logistics involved, the only practical option would be to transport cryopreserved sperm. Despite some early reports on African elephant semen cryopreservation, the utility of this new population management tool has not been evaluated.

**Methodology/Principal Findings:**

Semen was collected by electroejaculation from 14 wild African savanna elephant (*Loxodonta africana*) bulls and cryopreserved using the directional freezing technique. Sperm treatments evaluated included the need for centrifugation, the use of hen or quail yolk, the concentration of glycerol (3%, 5% or 7%) in the extender, and maintenance of motility over time after thawing. Our results suggest that dilution in an extender containing hen yolk and 7% glycerol after centrifugation best preserved post-thaw sperm motility when compared to all other treatments (*P≤*0.012 for all). Using this approach we were able to achieve after thawing (mean ± SD) 54.6±3.9% motility, 85.3±2.4% acrosome integrity, and 86.8±4.6% normal morphology with no decrease in motility over 1 h incubation at 37°C. Sperm cryopreserved during this study has already lead to a pregnancy of a captive female elephant following artificial insemination.

**Conclusions/Significance:**

With working techniques for artificial insemination and sperm cryopreservation of both African and Asian elephants in hand, population managers can now enrich captive or isolated wild elephant populations without removing valuable individuals from their natural habitat.

## Introduction

About 140 years ago, Charles Darwin wrote in his book *The Descent of Man, and Selection in Relation to Sex* the following prediction: “The slowest breeder of all known animals, namely the elephant, would in a few thousand years stock the whole world.” [1]. Unfortunately, primarily due to human activities, this prediction will probably not come true. Elephants under human care are not self-sustaining, not in zoos [2], [3] and, for different reasons, probably also not in their range countries [4]. The wild population is continually decreasing, even among African elephants, which are presumed to be fairing better [5]. Human dwellings that are constantly encroaching on elephant habitat, leading to human-elephant conflicts [6], [7] and fragmentation of populations [5] are bound to be on the rise, putting the wild population at ever growing risk. Small, isolated populations in the wild or under captive conditions face many risks; a major one of them is the risk of inbreeding and its consequential reduced fitness of the population. For example, a recent evaluation of the captive African elephant population in North America found an average heterozigosity of 0.53, significantly lower than the level reported for wild African elephants (0.64) [8]. One way to keep these populations’ gene pool sufficiently diverse is to continuously import new animals from the wild to replace old and deceased ones, something that has been in practice for many years but was considerably reduced since the Convention on International Trade in Endangered Species of Wild Fauna and Flora (CITES) was enacted in 1976. Another option is to establish a genome resource bank (GRB) for the three elephant species, in which gametes and tissues from genetically valuable individuals are stored and used to introduce genetic materials into captive and isolated wild populations as needed. Establishment of such banks, done prospectively and as a measure of gamete rescue, can serve to maintain high genetic diversity in relatively small populations as a result of the increase in generation time [9]. Several major steps in this direction have already taken place in recent years. These include the development of a technique to collect elephant semen [10] and accomplishment of successful artificial insemination in both Asian (*Elephas maximus*) and African savanna (*Loxodonta africana*) elephants [11], [12].

A major component of such GRB would be cryopreserved sperm. In the absence of techniques to collect the female elephant gametes or embryos *in vivo*, such sperm, being relatively simple to collect, can be the main mode of genetic material mobilization between populations. Attempts to cryopreserve African elephant sperm have been reported about 30–40 years ago [13], [14] but since then no further attempts to improve or verify those early studies were reported. Recently we have reported on a thorough study on the cryopreservation of Asian elephant spermatozoa, which has resulted in the development of a protocol for sperm cryopreservation in this species [15]. As in our study [15], other studies on Asian elephant sperm cryopreservation also found glycerol to be the cryoprotectant of choice and its addition after chilling to be preferential [16], [17].

Elephants in zoos are usually kept under very favorable conditions in terms of nutrition and health care. However, keeping males in the exhibits is a very demanding undertaking because of the extra space needed and costs incurred. Many zoos have therefore elected to keep only females and young offspring, resulting the current population male to female ratio of only about 1∶5.6 in North America and 1∶4.1 in Europe [18], [19], [20], [21]. Furthermore, only a fraction of these males actively participating in reproduction. Under natural circumstances, while female offspring stay with the maternal group, the males leave when they reach adulthood and either form small bachelor groups or wonder alone in search of mating opportunities [22], [23], [24]. Elephant bulls show a physiological and behavioral condition known as musth, which is exclusive to elephants [25], [26], [27]. Musth is manifested by bouts of elevated testosterone, secretion form the temporal glands, urine dribbling, heightened aggression and increased sexual activity [26], [28], [29]. Most observed matings are by males in musth, even when older and larger males in better body condition are present [26]. Thus, the phenomenon of musth changes the hierarchy between bulls. However, prolonged elevated testosterone damages sperm quality so that bulls in the height of their musth period actually cannot produce offspring. There is only a limited and short period of several days during which they can be highly reproductive [30].

The present study was designed to evaluate the possibility of adapting previously reported options to wild African savanna elephants’ sperm and importing such cryopreserved sperm for use in captive artificial insemination programs. Semen, collected from wild African savanna elephant bulls, was cryopreserved using different cryoprotective agents and concentrations thereof in search for the optimal combination. Cryopreserved semen was then stored under liquid nitrogen while samples underwent extensive virological and bacteriological examinations to preclude possible disease transmission. The ultimate goal of the study was to use the cryopreserved semen to introduce new genetic material into the zoo population in Europe and North America through artificial insemination.

## Materials and Methods

### 1. Ethics Statement

All procedures were conducted within the guidelines for IUCN CITES requirements and in accordance with the guidelines of the Internal Committee of Ethics and Animal Welfare of the Leibniz Institute for Zoo and Wildlife Research as approved under approval number 2009-08-01.

### 2. Animals

A total of 14 African savanna elephant (*Loxodonta africana*) bulls, ages 14 to 36, were collected for this study. All bulls lived in the Phinda Private Game Reserve, Republic of South Africa, which is located at S 27°46.657′ E 32°20.942′. Seven of the bulls were collected in the spring (September 2009), at the end of the dry season. Two of these and seven additional bulls were collected in the following fall (April 2010), at the end of the rainy season. In three cases, the same bull was collected twice in the same year.

### 3. Anesthesia

Following experience gained in previous projects [31], a capture gun (Parker Hale, Birmingham, UK) was used to deliver immobilizing drug mixture to the elephants. The dosage used, based on estimated body weight, was 12 to 20 mg of etorphine hydrochloride (M99, C-Vet Ltd., Bury St. Edmunds, United Kingdom) combined with 30 to 40 mg of azaperone (Stresnil®, Janssen-Cilag GmbH, Neuss, Germany). Anesthesia was reversed by intravenous administration of 26 to 42 mg of diprenorphine (Revivon M5050, C-Vet Ltd., Bury St. Edmunds, United Kingdom) in combination with 100 to 300 mg of naltrexone hydrochloride (Trexonil, Wildlife Pharmaceuticals, Fort Collins, CO), half of the dose administered intravenously and half intramuscularly.

### 4. Semen Collection and Evaluation

Semen was collected by electroejaculation (Seager model 14, Dalzell USA Medical Systems, The Plains, VA, USA) using a specially designed hand-held electrical probe. The position of the relevant organs was determined by preceding ultrasound. The design of the probe and water administered into the rectal lumen shortly before the start of stimulation enhanced electric coupling of the probe and facilitated reduction in the amount of voltage (15–30 V) and amperage (450–1000 mA) necessary for ejaculation. A total of 12–16 electrical stimuli were applied with increasing voltage and amperage. Each set of stimulations was followed by manual massage of the pelvic and penile aspects of the urethra. Collection bag placed over the penis funneled the semen into a foam insulated 50 mL vial located at the bottom of the bag. As a measure to reduce the risk of urine contamination, the urinary bladder and bladder neck were imaged by ultrasound so as to avoid their stimulation. Collection tubes were regularly exchanged as a further mean of caution against urinary contamination. Unless otherwise stated, semen samples were immediately diluted (1∶1) with pre-warmed (37°C), Berliner Cryomedium (BC) basic solution, which was modified for elephants [15]. The BC basic solution [32] is composed of 2.41% (w/v) TES, 0.58% (w/v) Tris, 0.1% (w/v) fructose and 5.5% (w/v) lactose, 15.6% (v/v) egg yolk and 20 IU α-tocopherol/mL. In the Japanese quail egg yolk treatment group, the hen egg yolk was replaced with quail egg yolk and this modified solution was used to dilute the sample and to make the freezing extender for this treatment group. The BC extender was chosen as standard sperm diluent over commercial extenders as it was shown to be effective in preserving Asian elephant semen [15] and semen from a variety of other species such as rhinoceros [33], European brown hare [34], or common hippopotamus [35].

Semen assessment upon collection included total volume (measured in a graded tube), sperm concentration estimated by Neubauer haemocytometer, and motility evaluated by dark-field microscopy (Olympus CX-41, Olympus Life Science Europe GmbH, Hamburg, Germany) using ×10 objective. The same experienced spermatologist evaluated all samples. Semen aliquots were fixed in Hancock’s fixative and were assessed for acrosome integrity and sperm morphology as previously described [15]. A total of 100 spermatozoa were counted per slide. Acrosomes were classified as intact versus modified or reacted (including completely detached acrosomes) [36]. Sperm morphology included search for a wide range of abnormalities as previously described [15].

### 5. Semen Cryopreservation

Semen freezing followed the same procedure we have previously reported for the Asian elephant [15]. During the first collection year, samples were tested using the following treatments in a split-sample design: (a) Diluted in BC basic solution with 3%, 5% or 7% glycerol (v/v) without centrifugation, (b) Centrifuged to remove seminal fluid and then diluted in BC basic solution with 5% glycerol, and (c) Diluted in BC basic solution with 5% glycerol in which the hen egg yolk was replaced with Japanese quail egg yolk. When volume was too small to split into all treatments, it was split into as many treatments as possible. In the second collection year, semen was cryopreserved following only one protocol - with centrifugation and BC extender containing 7% glycerol. When centrifugation was used, the samples were underlain with 2 mL of isothermal 60% iodixanol (OptiPrep™, Sigma–Aldrich Chemie GmbH, Taufkirchen, Germany) and centrifuged at ambient temperature (∼23°C) for 20 min at 1,000 g. The supernatant and OptiPrep were then aspirated and the pellet was resuspended in the freezing extender. Exclusion of centrifugation from some of the treatments was done with the aim of following previous attempts at freezing elephant sperm [14], [16]. All samples were diluted to a final concentration of 300 × 10^6^ cells/mL with extender containing 10% of the final glycerol volume. They were then cooled to 4–5°C at ∼0.3°C min^−1^ by submerging the tubes in isothermal water bath and placing it in a refrigerator. Once chilled, the samples were further diluted to 150 × 10^6^ cells/mL with isothermal extender containing the balance 90% of the glycerol to achieve the desired final glycerol concentration. Chilled samples were packaged into pre-chilled HollowTubes™ (IMT Ltd., Ness Ziona, Israel) of 2.5 mL (for later evaluation) and 8.0 mL (for storage) and frozen in a directional freezing machine (MTG-550, IMT Ltd., Ness Ziona, Israel) as previously described [15]. All frozen samples were stored under liquid nitrogen pending evaluation.

For evaluation, samples were thawed by first holding them in the air, at ambient temperature (∼23°C), for 90 s and then placing them into a specialized HollowTube™ thawing unit (IMT Ltd., Ness Ziona, Israel) inside a water bath at 37°C for 60 s. After thawing, samples were kept in a water bath at 37°C pending evaluation. Samples were evaluated after chilling and at three post-thaw time slots – immediately after thawing, and after 30 min and 60 min incubation in a 37°C water bath. Post-chilling and post-thaw evaluations included motility, acrosome integrity and sperm morphology as described above.

### 6. Health Assessment and Semen Importation

To facilitate importation, the game reserve was evaluated for the following transmissible diseases prior to collection: Rift Valley Fever, Foot and Mouth Disease, Rinderpest, Vesicular Stomatitis, Anthrax, Rabies, and Tuberculosis. Serum samples collected from each bull at the time of semen collection was also tested at an officially approved laboratory for Brucellosis, Foot and Mouth Disease, Rift Valley Fever and Bluetongue.

African elephants and their products are listed under appendix II of CITES and appendix B of the European Commission. To facilitate the importation of the semen samples, export and import CITES certificates were applied for.

### 7. Statistical Analysis

Statistical analysis was performed using IBM SPSS 19 (IBM Corporation, NY, USA). Separate analyses were performed for the acrosome, morphology and motility measurement variables. Values for motility, acrosome integrity and normal sperm morphology are reported as mean ± SD unless they are individual measurements (Table 1). Transformed percentage values based on the values of the native samples were used for statistical analysis. The treatments were compared using a Linear Mixed Model with the factor “treatment” (5 levels: 3% glycerol, 5% glycerol, 5% glycerol with centrifugation, 5% glycerol with quail egg yolk, 7% glycerol), as this model can cope with both missing values (due to limited volume of sperm) and double measurements (two bulls were caught in both years during the random sampling) in the data. In the motility analysis, a second factor “time” (random factor, 3 levels: 0 h, 0.5 h, 1 h) was included. In a first approach for each of the three measurement variables, the first collection year was analyzed and a pair-wise comparison of treatments (and of time points for motility) was performed (Sidak-adjustment). For motility, the comparison of treatment levels referred to the time factor as a whole, i.e., the analysis includes the three post thawing time levels simultaneously. Reversely, the comparison of time points referred to all treatments. In a second approach for each measurement variable, the second year was also included and the 7% glycerol with centrifugation treatment applied in the second year was compared with each treatments of the first year (Sidak-adjustment). A comparison of time points for the 7% glycerol with centrifugation treatment was added using a Repeated Measurements Analysis of Variance. Differences were considered significant when *P*<0.05.

**Table 1:** Native semen parameters of wild African elephant bulls collected during two consecutive years.

Bull No.	Age (y)	Total volume(mL)	Utilized volume(mL)	Concentration(10^6^/mL)	Motility(%)	Intact acrosome(%)*	Normal morphology (%)*
1^st^ year							
1a	35	11.1	11.1	562.5	84	88	78
2a	25	159.2	20.6	45.0	90	81	79
2b	25	43.1	21.6	1425.0	64	64	59
3a	17	68.5	68.5	1582.5	84	63	62
4a	17	156.6	131.6	220.0	87	33	31
4b	17	208.9	135.6	300.0	92	85	78
5	26	81.0	46.0	1134.0	93	52	47
6	30	4.5	4.5	1500.0	94	33	33
7	27	205.6	110.6	262.5	90	79	79
Mean ± SD	24.3±6.3	104.3±80.0	61.1±52.6	781.3±622.8	86.4±9.2	64.2±21.2	60.7±19.7
2^nd^ year							
1b	36	143.6	26.6	765.0	89	97	93
3b	18	97.3	64.0	375.0	82	97	77
8a	34	259.3	185.0	78.0	92	95	94
8b	34	67.3	13.3	735.0	95	93	92
9	25	57.3	46.7	2025.0	61	95	77
10	32	69.0	38.0	1000.0	90	94	94
11	26	23.7	23.7	787.5	89	91	66
12	14	54.6	38.0	1815.0	90	95	89
13	19	129.3	53.3	262.5	89	99	89
14	17	27.9	24.6	225.0	88	92	78
Mean ± SD	25.5±8.2	92.9±70.4	51.3±49.4	806.8±658.1	86.5±9.5	94.8±2.4	84.9±9.7

Letters next to the bull number indicate that the same bull was collected more than once either during the same year or in both years. Concentration relates to the utilized fraction and not to the total volume, which also included sperm-free or highly viscose portions that were discarded. Ages were estimated by the game reserve managers.

* Mean values were significantly different between the years for intact acrosome (*P* = 0.009) and normal morphology (*P* = 0.004).

## Results

Semen was successfully collected from all bulls with much variability between and within bulls in all evaluated parameters of the native semen - volume, concentration, motility, acrosome integrity and normal morphology (Table 1). In some bulls, portions of the ejaculates were either sperm-free or were too viscous for the spermatozoa to be separated by centrifugation. Such portions, which account for the difference between total volume and utilized volume in Table 1, were discarded.

**Figure 1:**
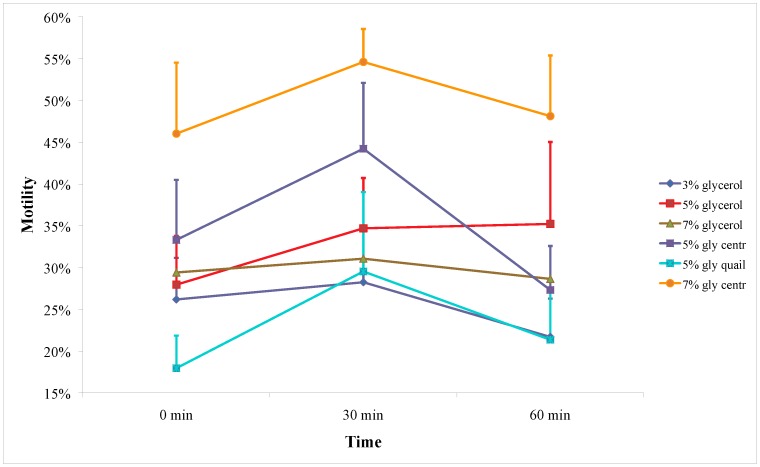
Wild African elephant sperm post-thaw motility at three different time points. Cryopreservation of African elephant sperm after discarding the seminal plasma by centrifugation and re-suspension in BC freezing extender with hen egg yolk and 7% glycerol resulted in significantly better post-thaw results as compared to all other treatments (*P≤*0.012 for all). Gly = glycerol; Centr = centrifuged; quail = with quail yolk. Error bars represent positive part of SD.

No differences for motility were found in the first collection year approach (treatments: *P = *0.099; time: *P = *0.187) or in any of the paired comparisons (see Supplementary material, tables S1 and S2). In the both collection years approach, with the 7% glycerol with centrifugation treatment included, significant differences between the treatments (*P*<0.001) were found. The 7% glycerol with centrifugation group resulted in 54.6±3.9% motile spermatozoa 30 min after thawing, significantly higher than each other treatment (3% glycerol: 28.2±6.6%, *P*<0.001; 5% glycerol: 34.7±6.0, *P* = 0.003; 5% glycerol with centrifugation: 44.2±7.9%, *P*<0.001; 5% glycerol with quail egg yolk: 29.5±9.5%, *P* = 0.012; 7% glycerol: 31.1±5.7%, *P*<0.001) (Figure 1). These comparisons between glycerol levels involve the time after thawing as a potentially influencing factor, but the results do not refer to a particular point of time. Furthermore, the time after thawing does not significantly influence the outcome of the experiments as no deterioration in motility during the 1 h post-thaw incubation at 37°C was found (*P* = 0.103) in the both years analysis. This was also true, when the analysis was restricted to the 7% glycerol with centrifugation treatment (*P = *0.555), the group with the best motility outcome. Evaluation of acrosome integrity and morphology measurements led to insignificant results in all comparisons of treatments for the analysis of the first collection year (acrosome integrity: *P = *0.737; morphology: *P = *0.715) and the respective paired comparisons (see Supplementary material, tables S3 for morphology and S4 for acrosome integrity). When the second year was included, the results were slightly significant as a whole (acrosome integrity: *P = *0.047; morphology: *P = *0.050), but not between any treatment from the first collection year and the 7% glycerol with centrifugation treatment of the second year, except the 5% glycerol with quail egg yolk treatment for morphology (see Supplementary material, table S3). Comparison of native sperm parameters between the two years found no difference in motility (*P* = 0.687) but significant differences in morphology (*P* = 0.009) and acrosome integrity (*P* = 0.004).

All elephants were confirmed negative for the diseases tested and the game reserve and its vicinity was shown to be free form Tuberculosis for the three years prior to semen collection, free from Rinderpest for at least 12 months, free from Vesicular Stomatitis, Rift Valley Fever and Rabies for at least six months, free from Foot and Mouth disease for at least 40 days and free from Anthrax for at least 30 days prior to semen collection.

## Discussion

The idea of tapping wild populations for material to increase the genetic diversity of captive or isolated populations is not new but has rarely been practiced [34], [37]. There are probably four main obstacles behind this apparent lack of utilization of such useful genetic resource: (i) Because of the distances involved, the only practical way of bringing semen from wild individuals for use in captive or isolated populations is by cryopreserving it. Protocols for semen cryopreservation are highly species-specific and for the vast majority of species such protocols still do not exist. Development of protocols can rely on knowledge accumulated in closely related species (usually domestic or laboratory animals) but eventually the protocol will have to be tested and modified on samples of the target species, a difficult undertaking when it comes to rare and endangered species; (ii) Assuming a proper semen cryopreservation technique is in hand, the next obstacle is lack of funds. The logistics involved in travelling of teams of experts, capturing the animals and anesthetizing them, collecting the semen, and cryopreserving the samples are very high and rarely available when dealing with wildlife; (iii) Again, assuming funds are raised and cryopreservation protocol is available, the collected samples will have to pass a large set of bacteriological and virological tests to make sure no disease is being transmitted. With all these tests and all needed permits (including export and import permits, and compliance with the Convention on Biological Diversity guidelines) in hand, samples can be transported to the target location; (iv) Once samples are in hand, the last obstacle is the development of successful artificial insemination procedures. To do that, a thorough understanding of the female’s reproductive cycle and related hormonal fluctuations as well as anatomical knowledge of its reproductive tract are needed. These may, at times, dictate the need to develop customized instruments. None of these obstacles is easy to scale. After many years of work invested in understanding the elephant cycle and the endocrinology involved and in developing techniques for semen collection, cryopreservation and artificial insemination, it was time to put things into practice. We are please to report that, at least in the case of the African savanna elephant, we were successful in showing that the concept is valid and working. As shown here, semen from wild African elephant bulls can be cryopreserved. The semen donors were checked for a variety of pathogens and, once confirmed negative, the semen was imported to Europe where it was used for AI [38]. While this pregnancy is of great importance, it is for now a single success. We will need to continue with our efforts and achieve more pregnancies to make the importation of semen and introduction of new genetic material into the population really effective. This process, though promising, is not without risks. A wide variety of pathogens can be transmitted in the sperm and thus a detailed screening test should be developed and enacted before such semen samples can be deemed safe for use. In the absence of clear and established guidelines and well-defined procedures, as nobody did this before, we had to work closely with the authorities to make the right and safe evaluation.

Current health legislations cover either the importation of semen samples collected from domestic species or the importation of whole wild animals but not the importation of semen samples collected from wild animals. Domestic species semen donors live in controlled environments and their health status is regularly and closely monitored. On the contrary, wild specimen range freely over large territories and their health status, including their status with regards to infectious disease, is not easily monitored. According to current legislations, only semen collected in approved collection and storage centers is eligible for importation. Importation of semen collected from wild animals is therefore a unique challenge from a health regulation point of view. A special import health certificate was therefore requested and has been issued to facilitate the importation of the samples. This health certificate is the end result of negotiations between the health authorities in Europe and the Republic of South Africa. This health certificate included a list of health requirement in accordance with the Republic of South Africa and Kwazulu-Natal district’s sanitary status, especially with regards to vector-borne diseases (Rift Valley Fever and Bluetongue) as well as Foot and Mouth Disease and Brucellosis, among others. Sanitary authorities consider the health risk assessment as being the same whether semen or live animals are being imported, although there are numerous limitations when dealing with semen collected in the wild from live animals. Access to the donor is limited to the collection process, follow-up examinations are not possible and tests such as antibodies kinetics are therefore not an option. A thorough clinical examination was performed on each semen donor and blood samples collected for disease screening at the time semen was collected. Microchips were inserted into each animal for identification purposes. All these allowed for a wide range of testing to be performed in order to provide health authorities with all available data that might be requested down the line. The health certificate includes three parts:

- Description of the samples or animals being imported (ID, origin, consignee etc.)
- Tests required and tests results
- Certificate of origin

As mentioned above, testing and results have some limitations when dealing with samples collected from wild animals. Another great challenge is to fulfill all requirements regarding the certificate of origin. When dealing with free ranging animals, epidemiological unit such as semen collection and storage center are no longer relevant and new ones have to be defined. Since the game reserve hosting the project is completely fenced, it has been accepted as an epidemiological unit and all requirements for sufficient time being disease-free of various pathogens were evaluated at the game reserve level.

African savanna elephant (*Loxodonta Africana*) and its products are listed on CITES appendix II and the European Commission appendix B. Export and Import CITES permits were therefore requested and have been issued in order to allow the importation of the samples.

Gamete cryopreservation in wildlife is done with the aim of finding the optimal protocol for each and every species under study as was done in the case presented here. Such studies considerably enhance our knowledge and enable us to set up cryopreserved semen collections or GRB’s. This, however, takes time, which is not always on our hands. People then resort to salvaging germplasm of deceased or neutered animals (e.g. [35], [39], [40], [41]). Gamete rescue activities are usually done in a rush, while the carcass is still fresh and viable gametes can still be retrieved. If successful, such attempts can extend the reproductive lifespan of these individuals and, through the use of their gametes, enhance the genetic management of the population. Species, however, are different from each other in many respects, one of which is the unique requirements their gametes have with respect to cryopreservation. Dealing with many species for which proper cryopreservation protocols are yet to be developed, efforts at gamete rescue, while enhancing our knowledge, often fail and the genetic material may be lost for the population.

Management of captive wildlife populations is restricted by the size of the groups within each zoological institution and this is indicated by the limited space available. To increase the genetic diversity and representation of important individuals in the population, animals are often moved between zoos. Transferring animals, however, is a stressful event to the individuals involved as well as to the herds at the origin and destination zoos. In elephants such translocation-induced stress was shown to increase the risk of mortality [42]. The alternative would be to mobilize gametes between institutions. Due to its size and the anatomic location of the ovaries and uterus within the female elephant body, *in vivo* oocyte or embryo collection in elephants is not practical and has never been reported. The only option is thus the use of semen collected in one institution to inseminate females at another. Several groups have been doing this since the development of artificial insemination in elephants about 15 years ago [11], [43]. While fresh chilled elephant semen can survive and maintain its fertilizing ability during transcontinental or even intercontinental transport, the need to use it fresh greatly limits its use. Thanks to the two precisely timed and spaced LH peaks in elephants [44], [45], [46], artificial insemination can be planned up to three weeks ahead of ovulation. Semen, however, can only be collected in proximity to ovulation time. For reasons not yet known, elephant bulls are extremely unpredictable in terms of the semen quality they produce, fluctuating between highly concentrated, viable and motile ejaculates and very poor, barely motile samples within a matter of days (Unpublished data; [47]). Unusable ejaculates can thus lead to missing the narrow window suitable for artificial insemination around ovulation. With ovulation occurring only once every four months or so in elephants, missing a cycle because of poor semen quality leads to long delay in conception. Use of cryopreserved semen, which can be available at any place and time and in predictable quality, can help overcome these limitations.

In this study we have attempted to prove this concept. A number of different treatments and extenders were evaluated in the search for the optimal cryopreservation protocol for African savanna elephant sperm. These included evaluation of the need for centrifugation to remove the seminal fluids, use of hen versus quail yolk in the extender and the concentration of glycerol as the cryoprotective agent. Due to constraints indicated by the conditions in the field and availability of sufficient samples, not all treatments could be conducted simultaneously on all samples in a true split-sample fashion. Thus, although the statistical analysis clearly indicated that the optimal cryopreservation protocol include centrifugation to remove the seminal fluids and then dilution in BC extender with hen egg yolk and 7% glycerol, a certain degree of uncertainty remains, because the time (season) of collection or the different bulls sampled rather than the extender might stand behind the differences found. Laws [48] proposed seasonal variations in male reproductive organs based on their data from culled elephants. However, Hanks and McIntosh [49] commented on Laws’ seasonality proposition saying that Laws’ results might be an artifact consequential of age differences between study groups. Seasonal changes along the reproductive tract, including the testes and accessory glands, are known in many seasonal animals (e.g. [50], [51]). If the elephant is a seasonal animal by nature, such a shift might be found when the same individuals are evaluated in consecutive seasons. The fact that differences in native sperm morphology and acrosome integrity were found between the two collection years suggest that at least in the sampled group of elephant, collection at the end of the rainy season might produce better quality sperm as compared to sperm collected at the end of the dry season. Our experience with the Asian elephant sperm [15] and our success in using this cryopreserved sperm in artificial insemination of a female elephant in captivity [38] suggest that the superior cryopreservation technique combined with better sperm quality collectively contributed to the fertile good quality sperm obtained after thawing.

We may conclude that cryopreservation of African elephant sperm can be done, and with good post-thaw results, when the ejaculated samples are centrifuged to remove the seminal fluids, diluted in BC extender containing hen egg yolk and 7% glycerol, and cryopreserved by the directional freezing technique. As a proof of the post-thaw functionality of spermatozoa cryopreserved using this protocol, we have reported recently on the first pregnancy in elephants using cryopreserved semen of a wild bull to inseminate a cow in captivity [38].

## Supporting Information

Table S1 Test for differences between treatments or time after thawing for motility in the first collection year.
(DOC)

Table S2 Pair-wise comparisons between treatments during the first collection year.
(DOC)

Table S3 Morphology values compared between the 7% glycerol with centrifugation treatment and each of the other treatments.
(DOC)

Table S4 Acrosome integrity values compared between the 7% glycerol with centrifugation treatment and each of the other treatments.
(DOC)
