# Supplementary material for: Freezing African Elephant Semen as a New Population Management Tool
Source: PLoS One. 2013 Mar 6;8(3):e57616. doi: 10.1371/journal.pone.0057616 (PMC3590205; doi:10.1371/journal.pone.0057616)
Supplement: Table S1 — Test for differences between treatments or time after thawing for motility in the first collection year. (DOC) [file pone.0057616.s001.doc]

**Table S1**: Test for differences between treatments or time after thawing for motility in the first collection year

| Factor | DF | F-value | Significance |
| --- | --- | --- | --- |
| Intercept | 1 | 345.600 | 0.000 |
| Glycerol | 4 | 2.114 | 0.099 |
| Time post thawing | 2 | 1.726 | 0.187 |
