# Supplementary material for: Freezing African Elephant Semen as a New Population Management Tool
Source: PLoS One. 2013 Mar 6;8(3):e57616. doi: 10.1371/journal.pone.0057616 (PMC3590205; doi:10.1371/journal.pone.0057616)
Supplement: Table S2 — Pair-wise comparisons between treatments during the first collection year. (DOC) [file pone.0057616.s002.doc]

**Table S2**: Pair-wise comparisons between treatments during the first collection year

| Treatment (I) | Treatment (J) | Significance |
| --- | --- | --- |
| 3% | 5% | 0.926 |
|  | 5% quail | 0.992 |
|  | 5% centr | 0.590 |
|  | 7% | 0.981 |
| 5% | 3% | 0.926 |
|  | 5% quail | 0.448 |
|  | 5% centr | 1.000 |
|  | 7% | 1.000 |
| 5% quail | 3% | 0.992 |
|  | 5% | 0.448 |
|  | 5% centr | 0.167 |
|  | 7% | 0.571 |
| 5% centr | 3% | 0.590 |
|  | 5% | 1.000 |
|  | 5% quail | 0.167 |
|  | 7% | 0.996 |
| 7% | 3% | 0.981 |
|  | 5% | 1.000 |
|  | 5% quail | 0.571 |
|  | 5% centr | 0.996 |

Centr = centrifugation, quail = quail yolk.
