# Supplementary material for: Freezing African Elephant Semen as a New Population Management Tool
Source: PLoS One. 2013 Mar 6;8(3):e57616. doi: 10.1371/journal.pone.0057616 (PMC3590205; doi:10.1371/journal.pone.0057616)
Supplement: Table S3 — Morphology values compared between the 7% glycerol with centrifugation treatment and each of the other treatments. (DOC) [file pone.0057616.s003.doc]

**Table S3**: Morphology values compared between the 7% glycerol with centrifugation treatment and each of the other treatments

| Morphology (I) | Morphology (J) | Significance |
| --- | --- | --- |
| 3% | 7% centr | 0.156 |
| 5% | 7% centr | 0.731 |
| 7% | 7% centr | 0.077 |
| 5% quail | 7% centr | 0.012 |
| 5% centr | 7% centr | 0.138 |

Centr = centrifugation, quail = quail yolk.
