# Supplementary material for: Freezing African Elephant Semen as a New Population Management Tool
Source: PLoS One. 2013 Mar 6;8(3):e57616. doi: 10.1371/journal.pone.0057616 (PMC3590205; doi:10.1371/journal.pone.0057616)
Supplement: Table S4 — Acrosome integrity values compared between the 7% glycerol with centrifugation treatment and each of the other treatments. (DOC) [file pone.0057616.s004.doc]

**Table S4**: Acrosome integrity values compared between the 7% glycerol with centrifugation treatment and each of the other treatments

| Acrosome (I) | Acrosome (J) | Significance |
| --- | --- | --- |
| 3% | 7% centr | 0.080 |
| 5% | 7% centr | 0.920 |
| 7% | 7% centr | 0.671 |
| 5% quail | 7% centr | 0.105 |
| 5% centr | 7% centr | 0.484 |

Centr = centrifugation, quail = quail yolk.
